# MiR-30c-5p regulates adventitial progenitor cells differentiation to vascular smooth muscle cells through targeting OPG

**DOI:** 10.1186/s13287-020-02127-2

**Published:** 2021-01-19

**Authors:** Qing Zhang, Ting Chen, Yun Zhang, Lingxia Lyu, Bohuan Zhang, Chengchen Huang, Xuhao Zhou, Yutao Wu, Zhoubin Li

**Affiliations:** 1 Department of Cardiology, The First Affiliated Hospital, Zhejiang University School of Medicine, Hangzhou, 310003 Zhejiang Province P.R. China; 2 Department of Lung Transplantation, The First Affiliated Hospital, Zhejiang University School of Medicine, Hangzhou, 310003 Zhejiang Province P.R. China

**Keywords:** MiR-30c-5p, OPG, Vascular progenitor cells, Vascular smooth muscle cells, Differentiation

## Abstract

**Background:**

As the most important component of the vascular wall, vascular smooth muscle cells (VSMCs) participate in the pathological process by phenotype transformation or differentiation from stem/progenitor cells. The main purpose of this study was to reveal the role and related molecular mechanism of microRNA-30c-5p (miR-30c-5p) in VSMC differentiation from adventitial progenitor cells expressing stem cell antigen-1(Sca-1).

**Methods:**

In this study, we detected the expression of miR-30c-5p in human normal peripheral arteries and atherosclerotic arteries. In vitro, a stable differentiation model from adventitial Sca-1^+^ progenitor cells to VSMCs was established using transforming growth factor-β1 (TGF-β1) induction and the expression of miR-30c-5p during the process was observed. Then, we explored the effect of miR-30c-5p overexpression and inhibition on the differentiation from adventitial Sca-1^+^ progenitor cells to VSMCs. The target genes of miR-30c-5p were identified by protein chip and biological analyses and the expression of these genes in the differentiation process were detected. Further, the relationship between the target gene and miR-30c-5p and its effect on differentiation were evaluated. Finally, the co-transfection of miR-30c-5p inhibitor and small interfering RNA (siRNA) of the target gene was implemented to verify the functional target gene of miR-30c-5p during the differentiation from adventitial Sca-1^+^ progenitor cells to VSMCs, and the dual-luciferase reporter gene assay was performed to detect whether the mRNA 3′untranslated region (UTR) of the target gene is the direct binding site of miR-30c-5p.

**Results:**

The expression of miR-30c-5p in the human atherosclerotic arteries was significantly lower than that in the normal arteries. During the differentiation from adventitial Sca-1^+^ progenitor cells to VSMCs, the expression of VSMC special markers including smooth muscle α-actin (SMαA), smooth muscle-22α (SM22α), smooth muscle myosin heavy chain (SMMHC), and h1-caponin increased accompanied with cell morphology changing from elliptic to fusiform. Meanwhile, the expression of miR-30c-5p decreased significantly. In functional experiments, overexpression of miR-30c-5p inhibited SMαA, SM22α, SMMHC, and h1-caponin at the mRNA and protein levels. In contrast, inhibition of miR-30c-5p promoted the expression of SMαA, SM22α, SMMHC, and h1-caponin. The target gene, osteoprotegerin (OPG), was predicted through protein chip and bioinformatics analyses. Overexpression of miR-30c-5p inhibited OPG expression while inhibition of miR-30c-5p had an opposite effect. Co-transfection experiments showed that low expression of OPG could weaken the promotion effect of miR-30c-5p inhibitor on the differentiation from adventitial Sca-1^+^ progenitor cells to VSMCs and the dual-luciferase reporter gene assay demonstrated that miR-30c-5p could target the mRNA 3′UTR of OPG directly.

**Conclusions:**

This study demonstrates that miR-30c-5p expression was significantly decreased in atherosclerotic arteries and miR-30c-5p inhibited VSMC differentiation from adventitial Sca-1^+^ progenitor cells through targeting OPG, which may provide a new target for the treatment of VSMCs-associated diseases.

## Background

Vascular smooth muscle cells (VSMCs) are the major components of the vascular wall and play a critical role in the development and progression of atherosclerosis (AS) and other cardiovascular diseases, such as stent restenosis, transplanted vascular restenosis, and hypertension [1]. Sca-1^+^ progenitor cells have been shown to exist in the adventitia of the vascular wall and to participate in the pathological process of vascular remodeling through migration and differentiation into VSMCs [2]. It has been shown that many factors, such as hyperlipidemia, dkk3, and stimulants, promote the migration process of the adventitial Sca-1^+^ progenitor cells [3, 4]. However, few studies have focused on the regulatory mechanism of adventitial Sca-1^+^ progenitor cell differentiation into VSMCs. Understanding the transcriptional regulatory circuitry of VSMC differentiation is essential to prevent these cardiovascular diseases and may prove useful for developing stem cell therapies.

MicroRNAs (miRs) are small non-coding RNAs about 22 nucleotides (22 nt) in length, and have been confirmed to be related to embryonic development, stem/progenitor cell differentiation, and other physiological and pathological processes [5, 6]. Mechanistically, miRs function as antisense regulators of their target genes by binding to the 3′untranslated region (UTR) of mRNAs [7–9]. Multiple miRs have been reported to play critical roles in the self-renewal and differentiation processes of stem cells. Our previous studies have shown that miRs, including miR-22, miR-34, miR-29, and miR-214 play essential roles in regulating VSMC differentiation from stem cells [10, 11]. MiR-30c-5p, a member of the miR-30 family containing miR-30a, miR-30b, miR-30c, miR-30d, and miR-30e, has been extensively shown to play a protective role in multiple cancers through inhibiting the proliferation and invasion abilities of tumor cells and increasing the sensitivity of tumor cells to chemotherapy. Moreover, miR-30c-5p has also been shown to participate in the processes of lipid metabolism, cell apoptosis, and epithelial to mesenchymal transition (EMT) associated with cardiovascular diseases, such as AS, hyperlipidemia, and hypertension in previous study [12]. However, none of these studies have investigated the role of miR-30-5p in the differentiation from adventitial Sca-1^+^ progenitor cells to VSMCs.

## Methods

### Collection of arterial specimens and clinical information

The healthy artery (HA) specimens were taken from the internal mammary arteries of patients aged 30 to 85 who underwent coronary artery bypass grafting at the First Affiliated Hospital of Zhejiang University (China) between June 2013 and August 2017. Diseased artery (DA) specimens were collected from patients who underwent selective lower limb amputation due to atherosclerotic occlusion. Exclusion criteria for the enrollment included liver failure, renal failure requiring dialysis treatment, tumors, pregnancy, and the lack of informed consent to participate in this study. In addition, we also collected and summarized the clinical characteristics including demographics, comorbidities and therapeutic drugs, and the hematological parameters of participants. All patients gave their written, informed consent prior to sample collection. All procedures were carried out following the rules of the Declaration of Helsinki and were approved by the Research Ethics Committee of the First Affiliated Hospital of Zhejiang University (Institutional Review Board approval No. 2013/150).

### Isolation and differentiation of adventitial Sca-1^+^ progenitor cells

Adventitial Sca-1^+^ progenitor cells were isolated from arteries of C57BL/6 mice (Shanghai Institutes for Biological Sciences, China) according to a previous study [2]. All animal experiments were conducted according to the ARRIVE guidelines. All animal procedures were carried out in accordance with the National Institutes of Health Guide for the Care and Use of Laboratory Animals. Briefly, the aortic arch, the aortic root, and part of the heart connecting to the aorta were removed and the adventitial tissues were dissected under the microscope. The adventitial tissues were cut into 0.5 mm pieces and transplanted onto a sliding chamber bottle inverted in a CO_2_ incubator for 3 h at 37 °C. The bottle was turned up and the cells were cultured with stem cell culture medium (CM) containing 10% fetal bovine serum (FBS: 10099141, Thermo Fisher Scientific, Waltham, MA, USA), leukemia inhibitory factor (10 ng/ml) (LIF: ESG1107, EMD Millipore, Burlington, MA, USA), and 2-mercaptoethanol (0.1 mM) (2-ME: M6250, Sigma Aldrich, St. Louis, MO, USA) for further microbead sorting. Cells collected using 0.25% trypsin were incubated with Sca-1 FITC (130-092-529, Miltenyi Biotec, Germany) for 10 min at 2–8 °C and incubated with Anti-FITC beads for another 15 min. The cells were put into a column surrounded by a magnetic cell sorting system (MACS). When the cell suspension was filtered, the Sca-1^+^ cells were collected and then cultured in a T25 bottle with CM. Cells sorted between passages 3 and 5 were induced to differentiate to VSMCs with the stimulation of differentiation medium (DM) containing TGF-β1 (10 ng/ml) (P04202, R&D Systems, Minneapolis, MN, USA), 10% FBS, and 2-ME (0.1 mM).

### Real-time quantitative polymerase chain reaction (RT-qPCR)

Total mRNA isolation and RT-qPCR were performed as we described previously [13]. Total RNA containing miRNAs was isolated from cells/tissues using Trizol (12183555, Invitrogen, Carlsbad, CA, USA) according to the manufacturer’s instructions. Total RNA- and miRNA-specific cDNA syntheses were performed using a PrimeScript RT Master Mix (Perfect Real-Time) Kit (RR047A, Takara, Japan) and a miDETECT A Track^TM^ miRNA qRT-PCR Starter Kit (C10712-1, Ribobio, China), respectively. Real-time PCR for mRNA was performed using Takara premix Ex Taq II (DRR820A, Takara, Japan) and was ran on an ABI Prism 7500 system (Applied Biosystems, Foster City, CA, USA) in a total volume of 10 μL containing 5 μL Takara premix Ex Taq II and 5 ng cDNA template. Real-time PCR for miRNA was performed with miDETECT A Track^TM^ miRNA qRT-PCR Starter Kit (C10712-1, Ribobio, China) and was ran on the same system in a total volume of 20 μL containing 10 μL SYBR Green Mix and 10 ng cDNA template. The PCR thermal cycling parameters for mRNA were 2 min at 50 °C, 30 s at 95 °C, 40 cycles of 95 °C for 5 s, and 60 °C for 34 s. For miRNA, the PCR parameters were 10 min at 95 °C and 40 cycles of 95 °C for 2 s, 60 °C for 20 s, and 70 °C for 10 s. Expression of mRNA/miRNA was normalized to the expression of mouse GAPDH (mGAPDH)/U6 and quantified using the 2^−ΔΔCq^ method. Primers used in the experiments are listed in Table 1.

**Table 1:** The sequence of primers used in the experiments

Gene	Forward (5′-3′)	Reverse (5′-3′)
GAPDH	AAACGGCTACCACATCCAG	CCTCCAATGGATCCTCGTTA
SMαA	TCCTGACGCTGAAGTATCCGAT	GGCCACACGAAGCTCGTTATAG
SM22α	GATATGGCAGCAGTGCAGAG	AGTTGGCTGTCTGTGAAGTC
SMMHC	AAGCAGCCAGCATCAAGGAG	AGCTCTGCCATGTCCTCCAC
h1-calponin	GGTCCTGCCTACGGCTTGTC	TCGCAAAGAATGATCCCGTC
OPG	ACCCAGAAACTGGTCATCAGC	CTGCAATACACACACTCATCACT
U6	CTCGCTTCGGCAGCACA	AACGCTTCACGAATTTGCGT
hsa-miR-30c-5p	GCGCGTGTAAACATCCTACACT	AGTGCAGGGTCCGAGGTATT
mmu-miR-30c-5p	GGCGTAAACATCCTACACTCTCAGC	miDETECT A Track™ miRNA qRT-PCR Starter

### Transfection of miRNAs, siRNAs, and plasmids

SiRNAs (80 nM, Baiao, China), miRNAs (mimic control/miR-30c-5p mimic: 20 nM; inhibitor control/miR-30c-5p inhibitor: 80 nM, Baiao, China), and plasmids (0.6μg/mL, NOVOBIO, Shanghai, China) were transfected into cells using Lipofectamine 3000 (L3000-015, Thermo Fisher Scientific, USA) following the manufacturer’s instructions. Briefly, when cells planted in 6-well plates reached 60–70% confluency, the medium was replaced with 900 μL empty media without antibiotics and maintained for 30 min before transfection. The cells were transfected with siRNAs (mixture of A and B; A: 50 μL DMEM/well + 4 μL siRNA/well; B: 50 μL DMEM/well + 3.75 μL Lipofectamine 3000/well), miRNAs (mixture of A and B; A: 50 μL DMEM/well + 1/4 μL miRNA/well; B: 50 μL DMEM/well + 3.75 μL Lipofectamine 3000/well), or plasmids (mixture of A and B; A: 50 μL DMEM/well + 0.6 μg plasmid/well; B: 50 μL DMEM/well + 3.75 μL Lipofectamine 3000/well) for 6 h. The cells were cultured for an additional 48 or 72 h.

### Western blot analysis

Protein extraction and immunoblotting were performed as described in our previous report [13]. Briefly, total proteins extracted using RIPA lysis buffer (P0013B, Beyotime, China) and quantified using a BCA Protein Assay Kit (P0012S, Beyotime, China) were boiled for 10 min at 100 °C. Ten to 30 μg of the proteins were separated in 10% SDS-PAGE gel (P0012A, Beyotime, China) and transferred from the gel to a membrane. The membrane was blocked overnight in 5% skimmed milk followed by incubation with primary antibodies against GAPDH (1:2000, 14C10, Cell Signaling Technology, Danvers, MA, USA), SMαA (Rabbit, 1:1000, 14395-1-AP, Proteintech, Rosemont, IL, USA), SM22α (Rabbit, 1:1000, Ab14106, Abcam, UK), SMMHC (Rabbit, 1:1000, AHP1117, AbD Serotec, UK), h1-calponin (Rabbit, 1:1000, Ab78491, Abcam, UK), and OPG (Rabbit, 1:1000, NB100-56505SS, Novus Biologicals, Centennial, CO, USA) overnight at 4 °C. The membrane was washed thrice with 1X PBST, for 10 min per wash, and incubated with the secondary antibody from Santa Cruz Biotechnology (Dallas, TX, USA) for 1 h at room temperature. The membrane was washed an additional 3 times with 1X PBST, the ECL substrate was added, and the target proteins were observed using the compact X4 in the dark room.

### Immunofluorescent staining

When the cells transfected with mimic control/miR-30c-5p mimic/inhibitor control/miR-30c-5p inhibitor were 70–80% confluent, they were fixed with 4% paraformaldehyde. Antibodies against SMαA (1:100), SM22α (1:250), SMMHC (1:50), and h1-calponin (1:150) were incubated with the fixed cells overnight at 4 °C followed by incubation with the fluorescent-labeled secondary antibody (SA00013-2/SA00013-4, Proteintech, Rosemont, IL, USA) for 1 h. The nuclei were stained with 4′,6-diamidino-2-phenylindole (DAPI: C1002, Beyotime, China) for 5 min before being photographed by immunofluorescence microscope and analyzed using Image J.

### VSMC proliferation and migration assays

To further confirm the role of miR-30c-5p in the AS process, the proliferation and migration abilities of VSMCs derived from adventitial Sca-1^+^ progenitor cells were detected through Edu and wound healing assays. Briefly, cells seeded on gelatin-coated 24/6 well plates were incubated with DM for 72 h, followed by transfection with mimic control/miR-30c-5p mimic/inhibitor control/miR-30c-5p inhibitor for 6 h. The cells were then incubated with 50 μM Edu for 2 h at 37 °C. Four percent paraformaldehyde was used to fix the cells after washing twice with 1X phosphate-buffered saline (PBS). Edu-incorporating cells were detected with Cell-LightTM Edu Apollo 567 (C10310-1, RiboBio, China) and captured with a fluorescence microscope. For the wound healing assay, a linear scratch was drawn across the cells layer when the confluent Sca-1^+^ progenitor cell-derived VSMCs were transfected with mimic control/miR-30c-5p mimic/inhibitor control/miR-30c-5p inhibitor, we then washed the exfoliated cells and captured under the microscope at once and 24 h later. The pictures were analyzed with Image J, and the percentage of Edu-positive cells and the healing area were calculated.

### Protein chip sequencing and dual-luciferase reporter gene assay

Protein chip sequencing was performed on the RayBio platform using G Series Mouse Cytokine Antibody Array 4000 (GSM-CAA-4000, RayBiotech, Peachtree Corners, GA, USA). The main steps included glass slide drying, protein loading, blocking, incubation with biotinylated antibody, incubation with Cy3 equivalent dye-streptavidin, fluorescence detection, and GSM-CAA-4000-SW data analysis. Dual-luciferase reporter gene assay was performed as our previous study [14]. The luciferase reporter was built based on the psiCHECK2 vector and the complete 3′UTR/mutant 3′UTR (mut-3′UTR) of OPG were cloned into it. HEK-293T cells were co-transfected with the luciferase reporter plasmids (psiCHECK2-OPG-3′UTR/psiCHECK2-OPGmut-3′UTR) and mimic control/miR-30c-5p mimic/inhibitor control/miR-30c-5p inhibitor by using Lipofectamine 3000. Luciferase and renilla activity were detected with a Dual-luciferase Reporter Assay kit (PR-E1910, Promega, Wisconsin, USA). Relative luciferase activity was defined as the ratio of renilla activity to luciferase activity with that of the control set as 1.0.

### Statistical analysis

Data were expressed as mean ± SEM and analyzed using a two-tailed Student’s *t* test for two-group comparison. Comparisons of different groups were performed using one-way ANOVA followed by Tukey’s HSD multiple comparison post-hoc test. A value of *P* < 0.05 was considered statistically significant.

## Results

### Comparison of characteristics between the HA and DA groups

Forty-seven patients aged 33 to 85 were finally enrolled in the present study according the selection and exclusion criteria. Twenty-seven patients were enrolled in the HA group, while the other 20 patients were enrolled in the DA group. The clinical parameters and hematological characteristics comparison between HA group and DA group were displayed in the Table 2. There were no differences in heart rate (HR), systolic blood pressure (SBP), diastolic blood pressure (DBP), smoking, hypertension, diabetes, chronic kidney disease (CKD), and stroke occurrence between the HA and DA groups. The body mass index (BMI) of patients in the HA group was higher than that in the DA group (24.22 ± 0.56 vs 21.59 ± 0.74, *P* = 0.006). There were no statistical differences between the groups in the hematological parameters including total cholesterol (TC), triglycerides (TG), high-density lipoprotein (HDL), low-density lipoprotein (LDL), very low-density lipoprotein (VLDL), estimated glomerular filtration rate (eGFR), serum creatinine (SCr), and hemoglobin and medical treatment with aspirin, statins, angiotensin-converting enzyme inhibitors (ACEI)/angiotensin-receptor blockers (ARB), and antiplatelet drugs. However, the number of patients taking β-blockers in the HA group was significantly higher than in the DA group (19 vs 2, *P* = 0.000).

**Table 2:** Hematological and clinical parameters of patients in this study

Patient information	HA group (***n*** = 27)	DA group (***n*** = 20)	***P*** value
Male, no. (%)	23 (85.2)	19 (95.0)	0.377
Age (years)	62.56 ± 1.42	67.75 ± 3.26	0.156
BMI (kg/m^2^)	24.22 ± 0.56	21.59 ± 0.74	**0.006**
HR (bpm)	76.10 ± 2.44	82.25 ± 2.27	0.080
SBP (mmHg)	127.70 ± 2.81	128.50 ± 4.41	0.874
DBP (mmHg)	73.70 ± 1.83	75.85 ± 2.70	0.499
Current smoker, no. (%)	9 (33.3)	5 (25.0)	0.537
Past smoker, no. (%)	15 (55.6)	11 (55.0)	0.970
Hypertension, no. (%)	17 (63.0)	11 (55.0)	0.582
Diabetes, no. (%)	10 (37.0)	4 (20.0)	0.347
CKD, no. (%)	1 (3.7)	3 (15.0)	0.298
Past stroke, no. (%)	2 (7.4)	1 (5.0)	0.613
Aspirin, no. (%)	25 (92.6)	20 (100.0)	0.500
Statin, no. (%)	25 (92.6)	14 (70.0)	0.100
ACEI/ARB, no. (%)	12 (44.4)	5 (25.0)	0.170
Beta blockers, no. (%)	19 (70.4)	2 (10.0)	**0.000**
Antiplatelet drugs, no. (%)	23 (85.2)	11 (55.0)	0.056
Triglycerides (mM/L)	1.37 ± 0.81	1.15 ± 0.83	0.069
Total cholesterol (mM/L)	3.32 ± 0.15	3.62 ± 0.22	0.251
HDL cholesterol (mM/L)	0.85 ± 0.05	0.96 ± 0.09	0.319
LDL cholesterol (mM/L)	1.76 ± 0.12	1.83 ± 0.15	0.726
VLDL cholesterol (mM/L)	0.63 ± 0.48	0.69 ± 0.06	0.399
eGFR (ml/min)	79.09 ± 4.51	86.26 ± 9.48	0.463
SCr (μmol/L)	104.37 ± 20.06	110.50 ± 30.47	0.862
Hemoglobin (g/L)	127.30 ± 4.40	114.85 ± 5.86	0.090

### MiR-30c-5p expression was significantly downregulated in VSMC differentiation and in human diseased arteries

**Fig. 1:**
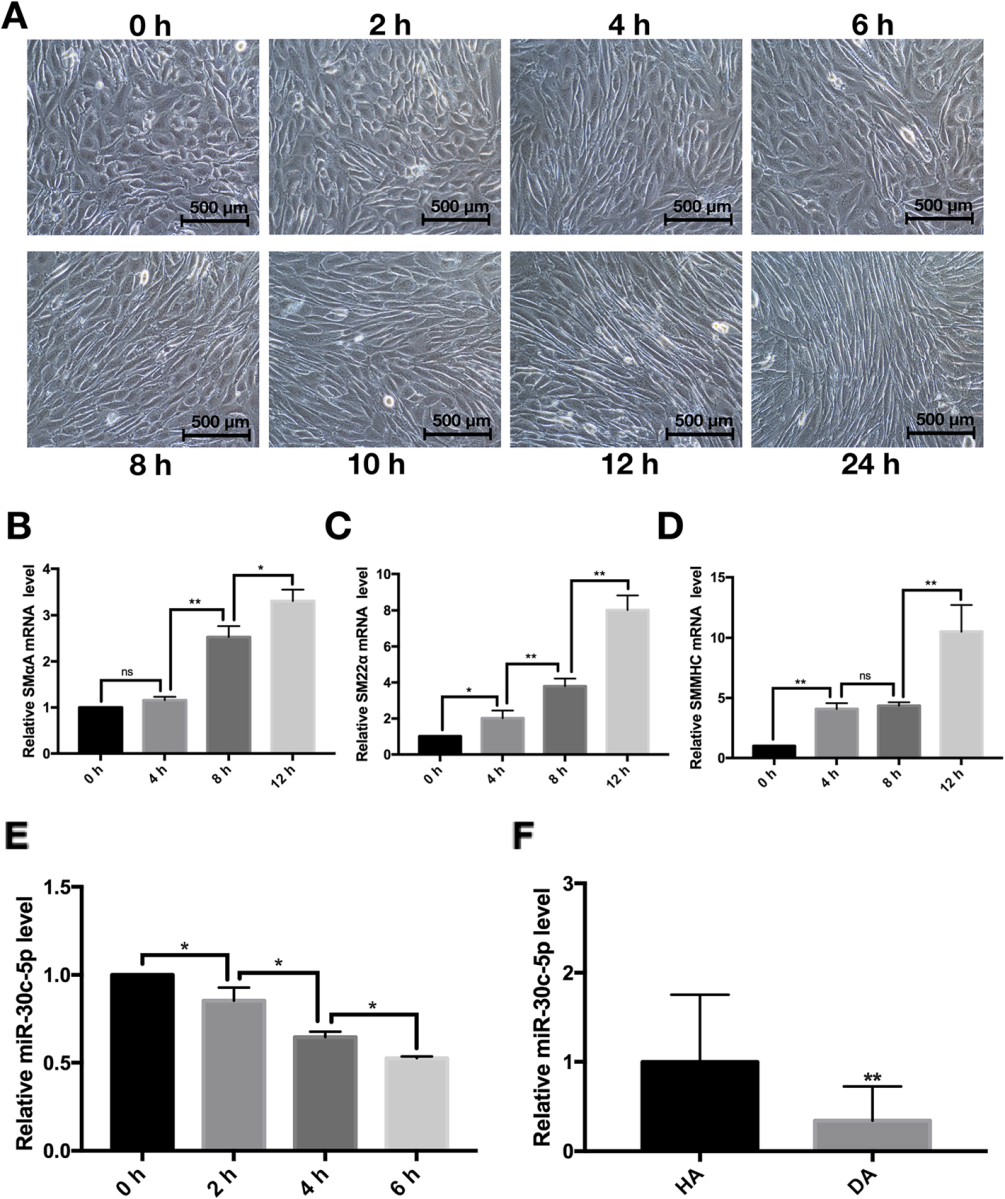
The change of miR-30c-5p in the process of VSMC differentiation and in DA. Adventitial Sca-1^+^ progenitor cells were induced by TGF-β1. The shape of the cells was observed under the microscope at different time points (0 h, 2 h, 4 h, 6 h, 8 h, 10 h, 12 h, 24 h) (**a**); the expression of SMαA (**b**), SM22α (**c**), SMMHC (**d**), and miR-30c-5p (**e**) were detected by RT-qPCR. The expression of miR-30c-5p in HA and DA were detected by RT-qPCR (**f**) (*P < 0.05, ** *P* < 0.01, ns *P* ≥ 0.05)

To induce VSMC differentiation, the adventitial Sca-1^+^ progenitor cells were seeded on 6-well plates and cultured with DM for 24 h. Consistently, the shape of the cells gradually changed from short ellipses to fusiform (Fig. 1a), accompanied with an obvious increase of VSMC-specific marker genes, including SMαA (Fig. 1b), SM22α (Fig. 1c), and SMMHC (Fig. 1d). Interestingly, the expression of miR-30c-5p was significantly decreased during the differentiation process (Fig. 1e). In addition, we also found a downtrend of miR-30c-5p in the human DA specimens versus HA specimens (Fig. 1f). These data suggested that miR-30c-5p may play an important role in the VSMC differentiation process as well as the process of AS.

### MiR-30c-5p inhibited VSMC differentiation in vitro

**Fig. 2:**
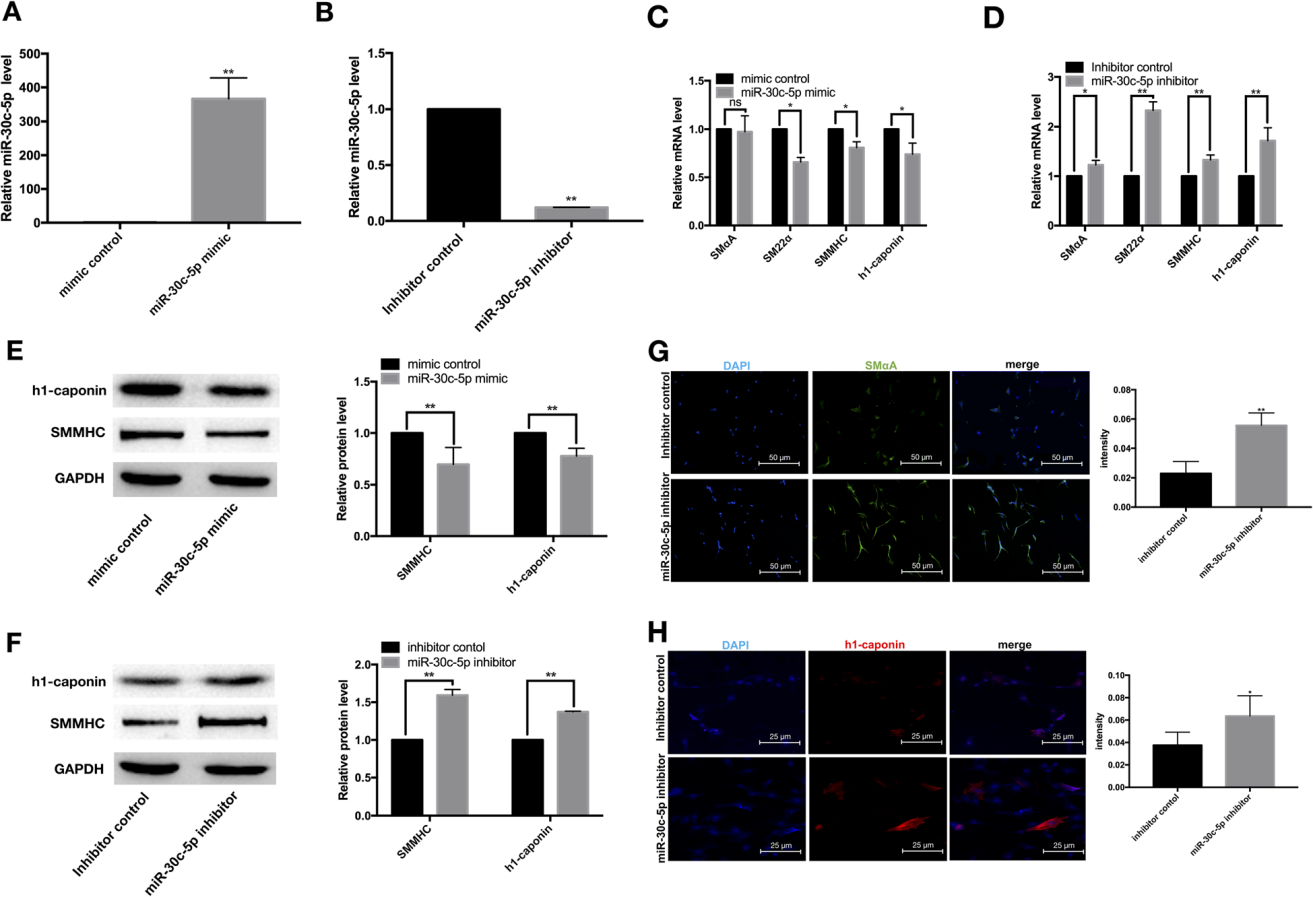
MiR-30c-5p inhibits VSMC differentiation from adventitial Sca-1^+^ progenitor cells. Adventitial Sca-1^+^ progenitor cells were transfected with mimic control/miR-30c-5p mimic/inhibitor control/miR-30c-5p inhibitor 4–6 h prior to TGF-β1 induced differentiation. The cells were collected after another 72 h. The transfection efficiency of miR-30c-5p mimic and miR-30c-5p inhibitor was detected by RT-qPCR (**a**, **b**). The expression of SMαA, SM22α, SMMHC, and h1-caponin were detected by RT-qPCR (**c**, **d**). The expression of SMMHC and h1-caponin were detected by western blot (**e**, **f**). The expression of SMαA and h1-caponin were detected by immunofluorescence (**g**, **h**) (*P < 0.05, **P < 0.01, ns *P* ≥ 0.05)

To investigate the role of miR-30c-5p during the differentiation from adventitial Sca-1^+^ progenitor cells into VSMCs, we detected the transfection efficiency of miR-30c-5p mimic/miR-30c-5p inhibitor and found that miR-30c-5p mimic increased the expression of miR-30c-5p in cells by about 400-fold (Fig. 2a), while miR-30c-5p inhibitor inhibited about 90% of miR-30c-5p in cells (Fig. 2b). The gain-of-function experiment using miR-30c-5p mimic showed that VSMC-specific markers SMαA, SM22α, SMMHC, and h1-calponin were decreased at the mRNA and protein levels (Fig. 2c and e). Simultaneously, the opposite effects were present in the loss-of-function experiment using miR-30c-5p inhibitor (Fig. 2d and f). Consistently, more SMαA-positive and h1-caponin-positive cells were presented in the miR-30c-5p inhibitor group as demonstrated by immunofluorescent staining and further Image J analysis (Fig. 2g and h). Taken together, these data firmly suggested that miR-30c-5p inhibits the differentiation of adventitial Sca-1^+^ progenitor cells into VSMCs.

### Effects of miR-30c-5p on the proliferation and migration of differentiated VSMCs

**Fig. 3:**
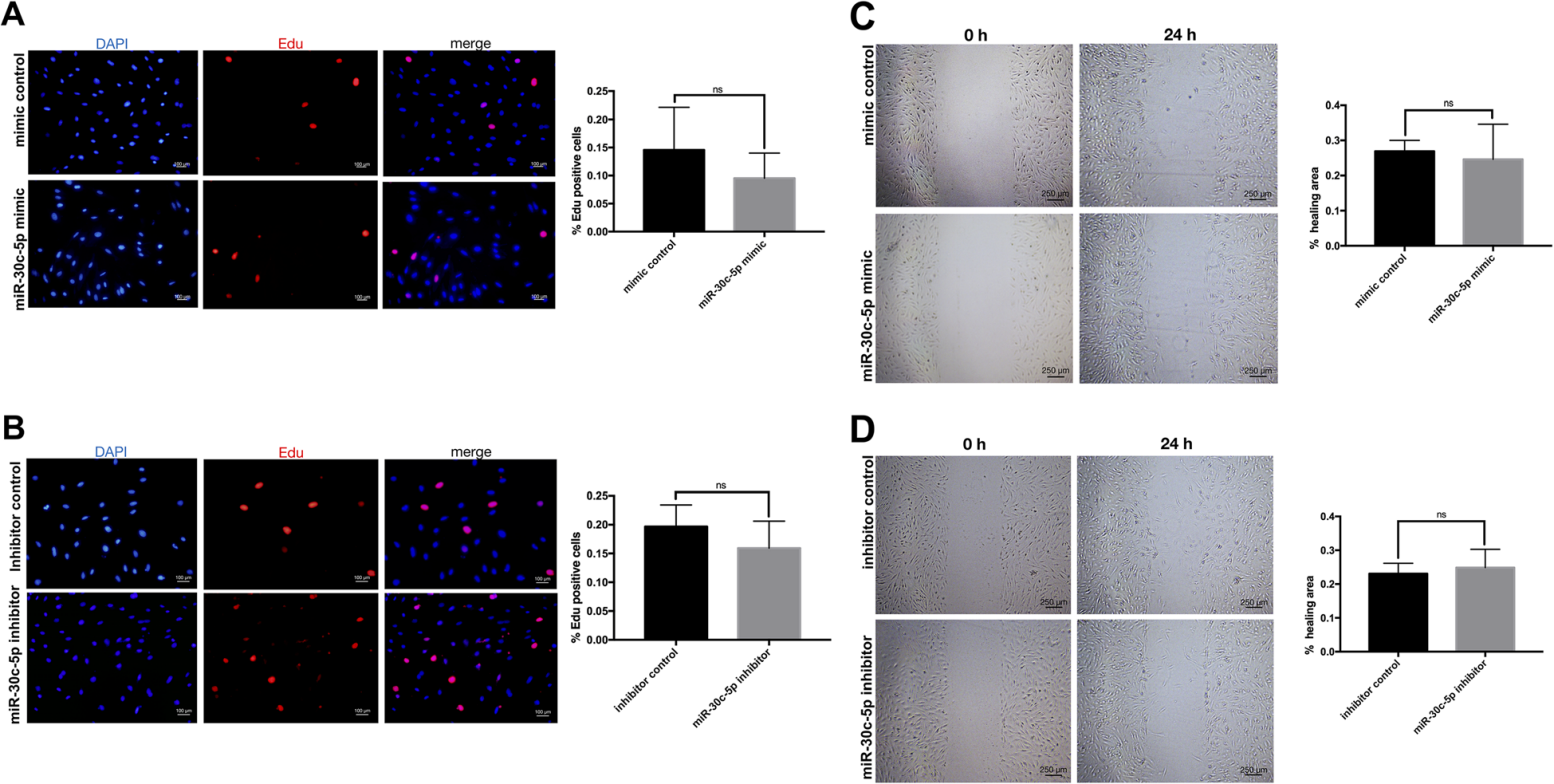
Effects of miR-30c-5p on the proliferation and migration of VSMCs derived from adventitial Sca-1^+^ progenitor cells. The VSMCs derived from adventitial Sca-1^+^ progenitor cells were transfected with mimic control/miR-30c-5p mimic/inhibitor control/miR-30c-5p inhibitor. The cells in the proliferative phase were detected by Edu experiment (**a**, **b**). The healing area was detected by the wound healing assay (**c**, **d**) (*P < 0.05, **P < 0.01, ns *P* ≥ 0.05)

As the most important component of the vascular wall, VSMCs participate in the AS process not only through stem/progenitor cell differentiation, but also through phenotypic transformation. When VSMCs undergo a phenotypic transition from a contractile phenotype to a migration and proliferation phenotype, the proliferation and migration abilities of them will obviously increase. Therefore, in addition to exploring the role of miR-30c-5p in regulating VSMC differentiation, we also investigated the effects of miR-30c-5p on the proliferation and migration of differentiated VSMCs through Edu and wound healing assays. Mimic control/miR-30c-5p mimic/inhibitor control/miR-30c-5p inhibitor was transfected into differentiated VSMCs, but we found there were no significant differences within 24 h in the percentage of Edu-positive cells (Fig. 3a and b) or the healing area (Fig. 3c and d) between the experimental group and the corresponding control group.

### OPG might be the target gene of miR-30c-5p during VSMC differentiation

**Fig. 4:**
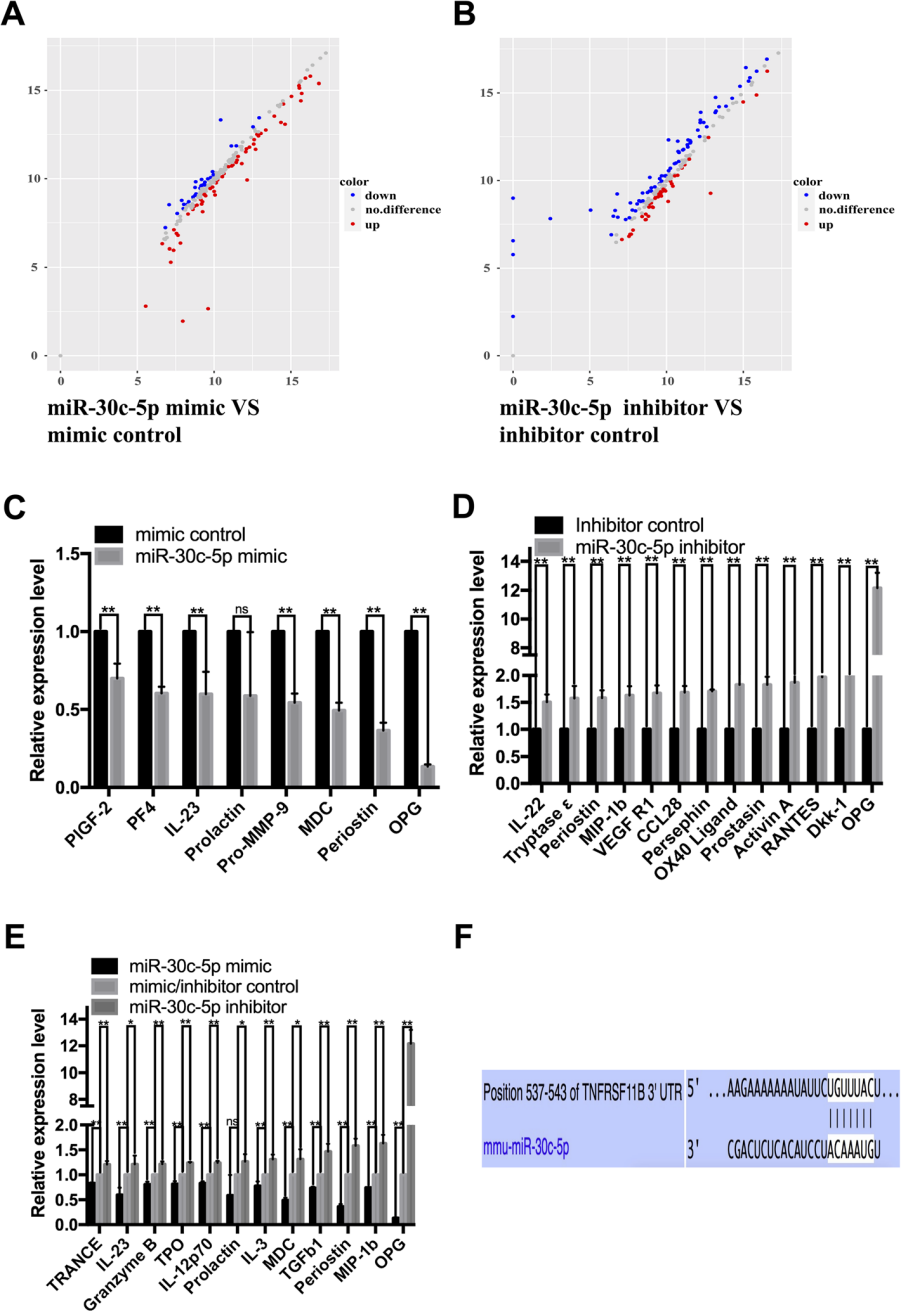
OPG may be the target gene of miR-30c-5p during VSMC differentiation from adventitial Sca-1^+^ progenitor cells. Adventitial Sca-1^+^ progenitor cells were transfected with mimic control/miR-30c-5p mimic/inhibitor control/miR-30c-5p inhibitor 4–6 h prior to TGF-β1 induced differentiation. Cells were collected for protein chip sequencing after another 72 h. Overall regulation of 200 proteins by miR-30c-5p mimic and miR-30c-5p inhibitor (**a**, **b**). Representative proteins downregulated (fold change < 0.667) by miR-30c-5p mimic (**c**) and representative proteins upregulated (fold change > 1.500) by miR-30c-5p inhibitor (**d**). The proteins that were both downregulated (fold change < 0.800) by miR-30c-5p mimic and upregulated (fold change > 1.200) by miR-30c-5p inhibitor (**e**). Target site of miR-30c-5p on the mRNA 3′UTR of TNFRSF11B (alias of OPG) (**f**) (*P < 0.05, **P < 0.01, ns *P* ≥ 0.05)

To explore the mechanism of miR-30c-5p involved in the differentiation process, the induced adventitial Sca-1^+^ progenitor cells transfected with mimic control/miR-30c-5p mimic/inhibitor control/miR-30c-5p inhibitor for 72 h were subjected to chip sequencing that included 200 proteins. MiR-30c-5p mimic and miR-30c-5p inhibitor upregulated part of the proteins (fold change > 1.200), while downregulated (fold change < 0.800) others (Fig. 4a and b). Since miRs induce the mRNA cleavage or translation suppression of their targets by binding to the 3′UTR of mRNAs, we further raised the selection criteria (upregulation: fold change > 1.500, downregulation: fold change < 0.667) and narrowed the screening range to screen out 8 factors downregulated by miR-30c-5p mimic and 13 factors upregulated by miR-30c-5p inhibitor (Fig. 4c and d). We also selected 12 possible target genes from the overlapping regions of the proteins downregulated (fold < 0.800) by miR-30c-5p mimic and the proteins upregulated (fold > 1.200) by miR-30c-5p inhibitor (Fig. 4e). The results showed that OPG was the best candidate for the target gene of miR-30c-5p. Furthermore, we found the target site of miR-30c-5p on the mRNA 3′UTR of TNFRSF11B (alias of OPG) according the TargetScan (Fig. 4f), a website predicting target genes of miRs. In summary, we predicted that OPG was a possible target gene of miR-30c-5p during the VSMC differentiation from adventitial Sca-1^+^ progenitor cells.

### miR-30c-5p inhibited VSMC differentiation through targeting OPG

**Fig. 5:**
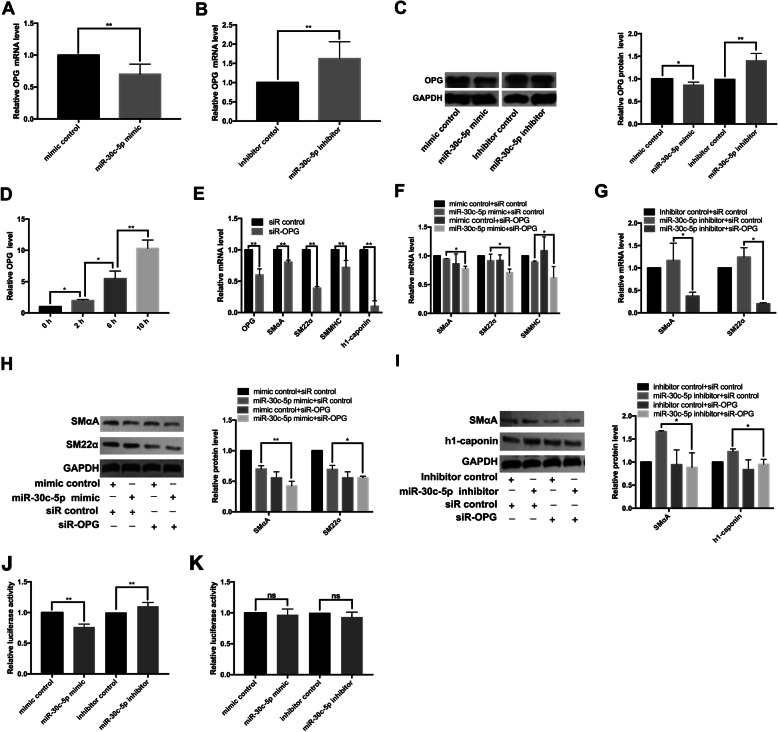
MiR-30c-5p inhibits VSMC differentiation from adventitial Sca-1^+^ progenitor cells through targeting OPG. Adventitial Sca-1^+^ progenitor cells were transfected with mimic control/miR-30c-5p mimic/inhibitor control/miR-30c-5p inhibitor for 12–24 h. The expression of OPG was measured by RT-qPCR (**a**, **b**) and western blot (**c**). Adventitial Sca-1^+^ progenitor cells were induced by TGF-β1. The expression of OPG was detected by RT-qPCR at different time points (0 h, 2 h, 6 h, 10 h) (**d**). Adventitial Sca-1^+^ progenitor cells were transfected with siRNA control and siRNA-OPG 4–6 h before the TGF-β1 induced differentiation and the cells were collected after another 72 h. The expressions of OPG, SMαA, SM22α, SMMHC, and h1-caponin were measured by RT-qPCR (**e**). Adventitial Sca-1^+^ progenitor cells were co-transfected with siR-OPG and miR-30c-5p mimic/miR-30c-5p inhibitor 4–6 h before the TGF-β1 induced differentiation and the cells were collected after another 72 h. The expression of SMαA, SM22α, SMMHC, and h1-caponin were detected by RT-qPCR and western blot (**f**–**i**). HEK-293T cells were co-transfected with psiCHECK2-OPG-3′UTR (**j**) or psiCHECK2-OPGmut-3′UTR (**k**) and mimic control/miR-30c-5p mimic/inhibitor control/miR-30c-5p inhibitor, and the luciferase and renilla activity were detected by luminometer and the relative luciferase activity was calculated (*P < 0.05, **P < 0.01, ns *P* ≥ 0.05)

To investigate whether OPG is a functional target gene through which miR-30c-5p regulates the differentiation of adventitial Sca-1^+^ progenitor cells to VSMCs, we measured the effect of miR-30c-5p on OPG by RT-qPCR and western blot analyses and found that miR-30c-5p mimic inhibited OPG expression at the mRNA and protein levels, while miR-30c-5p inhibitor had an opposite effect (Fig. 5a–c). We further measured the expression of OPG during the differentiation of adventitial Sca-1^+^ progenitor cells into VSMCs by RT-qPCR and found that the expression of OPG increased gradually (Fig. 5d), which is in contrary to the changing trend of miR-30c-5p during the process. We found that siR-OPG significantly reduced the expression of OPG and mimicked the inhibitory effects of miR-30c-5p mimic on SMαA, SM22α, SMMHC, and h1-caponin (Fig. 5e). Co-transfection of miR-30c-5p mimic and siR-OPG found that siR-OPG further reduced SMαA, SM22α, and SMMHC at mRNA level (Fig. 5f) and SMαA and SM22α at protein level (Fig. 5h), while co-transfection of miR-30c-5p inhibitor and siR-OPG showed that siR-OPG attenuated the promotion effect of miR-30c-5p inhibitor on SMαA and SM22α at mRNA level (Fig. 5g) and SMαA and h1-caponin at protein level (Fig. 5i). Dual-luciferase reporter gene assay with reporter plasmids (psiCHECK2-OPG-3′UTR/psiCHECK2-OPGmut-3′UTR) was performed to detect whether the mRNA 3′UTR of OPG was in response to miR-30c-5p and we found co-transfection of miR-30c-5p mimic and psiCHECK2-OPG-3′UTR reporter plasmids diminished the relative luciferase activity while co-transfection of miR-30c-5p inhibitor and psiCHECK2-OPG-3′UTR reporter plasmids had an opposite effect (Fig. 5j). As expected, the co-transfection of miR-30c-5p mimic/miR-30c-5p inhibitor and psiCHECK2-OPGmut-3′UTR had no impact on the relative luciferase activity (Fig. 5k). In summary, the data showed that miR-30c-5p regulated the differentiation of Sca-1^+^ progenitor cells into VSMCs by targeting OPG.

## Discussion

Excessive proliferation and aggregation of VSMCs are the main promoters of atherosclerotic plaques. In the past, it was thought that the increasing proliferative capacity of VSMCs induced by phenotype transformation from a contractile phenotype to a proliferative phenotype promoted the plaque formation. However, VSMC-related stem/progenitor cells including Sca-1^+^, CD34^+^, and c-kit^+^ cells in the “progenitor cell pool” have been shown to differentiate into VSMCs participating in AS [2]. In the present study, TGF-β1 was used to construct the VSMC differentiation model, which is more ethical compared with the model using embryonic stem cells and more rapid, efficient, and complete compared with the model induced by platelet derived growth factor BB (PDGF-BB) or collagen IV [15–17]. With TGF-β1 induction, the expression of VSMC-specific markers SMαA, SM22α, and SMMHC obviously increased and were accompanied by the cell morphology change from short ellipse to fusiform, which is consistent with the previous research results demonstrating that TGF-β1 could induce the Sca-1^+^ progenitor cells to differentiate into VSMCs [18].

The important role of miRs in regulating VSMC differentiation has been confirmed by the defects in contraction and differentiation of VSMCs in both the specific VSMC dicer knockout model and system dicer or miR-143/145 knockout models [19, 20]. MiR-30c-5p, a member of miR-30 family, has been extensively studied in the field of oncology. Many studies showed that miR-30c-5p was downregulated in multiple tumor tissues and inhibited the proliferation/migration of tumor cells and the neovascularization of the tumor tissues by targeting SOX9/TGF-β [21–23]. In addition, miR-30c-5p has also been shown to be involved in the regulation of myocardial fibrosis, myocardial infarction, aneurysms, and many pathological processes of cardiovascular diseases. In the present study, we found that the expression of miR-30c-5p in HA was significantly higher than in DA, which is consistent with the previous study on serum levels [24]. The negative correlation between miR-30c-5p and TG, LDL, carotid intima-media thickness, plaque development, and severity of coronary artery disease has been confirmed in a previous study [25], so we were able to assume that miR-30c-5p participates in the pathological process of AS. We also found that the BMI of patients in the HA group was higher than that in the DA group. One possible explanation is the limitation of the BMI index, in that it cannot distinguish not only the relative content of muscle and adipose tissue, but also the relative content of subcutaneous and visceral fat [26]. Another possible explanation is the “U” correlation between BMI and the burden of AS disease in which the BMI of two groups is at the symmetrical position of the bottom although statistical differences can be calculated [27, 28]. In addition, there were more patients receiving β-blocker treatment in the HA group than in the DA group, which may be explained by the pharmacological effects of β-blockers. β-blockers can slow heart rate and reduce myocardial oxygen consumption by inhibiting β1 receptors expressed on the myocardium. However, the hypotensive effect of β-blockers in arteriosclerotic occlusive disease can be replaced by many drugs.

MiR-30c-5p has been shown to inhibit the apoptosis of endothelial cells induced by NLRP3 and inhibit the proliferation or collagen production of cardiac fibroblasts through TGFβRII [29, 30]. MiR-30c-5p has also been proven to increase cell viability and inhibit the production of lactate dehydrogenase during myocardial ischemia-reperfusion injury [31]. Some studies have shown that miR-30c-5p played an important role in embryonic development and the differentiation of stem/progenitor cells [21]. However, there was no conclusion on whether miR-30c-5p is involved in VSMC differentiation. In this study, we found that the expression of miR-30c-5p gradually decreased during the differentiation process from adventitial Sca-1^+^ progenitor cells into VSMCs, suggesting a critical role in this process. We further found that the overexpression of miR-30c-5p inhibited the expression of VSMC markers SMαA, SM22α, SMMHC, and h1-caponin at the mRNA level, while miR-30c-5p knockdown had an opposite effect, which suggested that miR-30c-5p inhibits the differentiation of VSMCs. However, the more obvious changes were concentrated on SMMHC and h1-caponin at the protein level while the changes of SMαA and SM22α were less pronounced obvious. The possible reason is that we collected relatively advanced differentiated cells and SMαA and SM22α are relatively early-expressed proteins during VSMC differentiation. VSMCs with strong proliferation/migration ability transformed from contractile VSMCs are the other sources of VSMCs involved in vascular remodeling-related diseases such as AS [32]. In order to further explore the role of miR-30c-5p in AS, VSMCs differentiated from adventitial Sca-1^+^ progenitor cells were used to detect the effects of miR-30c-5p on their proliferation and migration capabilities. It was found that miR-30c-5p had no significant effect on the proliferation and migration of differentiated VSMCs within 24 h. However, the current results are not sufficient to conclude that miR-30c-5p does not participate in the regulation of phenotypic transformation of differentiated VSMCs and relevant experiments are needed to test the change of contraction ability, expression of contraction-related proteins, and ability to synthesize extracellular matrix of the VSMCs.

Several platforms including TargetScan, miRTarBase, miRanda, miRecords, and miRWalk predicting target genes of miRs have been developed. There are some differences in the prediction results because of different calculation methods, but false positives are present in all calculation methods. There were 5531 possible target genes of miR-30c-5p predicted by miRwalk and 1445 possible target genes predicted by TargetScan using the most classical (3′UTR) target sites. We also performed a protein chip experiment to assist in the screening of target genes and found the number of proteins downregulated by miR-30c-5p mimic was more than 30 and the number of proteins upregulated by miR-30c-5p inhibitor was more than 40. OPG was chosen to be the most suitable candidate for the target gene of miR-30c-5p through multiple screening methods including KEGG and GO enrichment analysis, raising screening criteria, intersection of proteins downregulated by miR-30c-5p mimic and proteins upregulated by miR-30c-5p inhibitor, and combining results predicted by protein chip sequencing and prediction platforms.

OPG, known as tumor necrosis factor receptor superfamily 11B (TNFRSF11B), is an important regulator of bone metabolism. Simonet et al. [33] found that osteoporosis occurred in OPG knockout animals, while overexpressed OPG induced osteopetrosis [34], which proved that OPG played a protective role in bone metabolism. In addition, OPG has also been shown to promote the growth and development of cancers by inhibiting the apoptosis of tumor cells through TNF-related apoptosis inducing ligand (TRAIL) and promoting neovascularization of tumor tissues through ERK [35–39], which is in contrast to the suppressive effect of its upstream molecule miR-30c-5p on tumors. Along with severe osteoporosis in OPG knockout mice, arterial calcification and aneurysms also appeared [34], attracting interest in OPG research in the cardiovascular field. As for the impact of OPG on AS, there was a paradox that overexpression OPG in a high cholesterol apoE knockout mouse model stabilized the AS plaques, while higher levels of OPG were detected in patients with AS than in corresponding controls in most clinical observational studies [40–44]. Some studies demonstrated that the expression of OPG was positively correlated with the thickness of artery intima and high levels of OPG could be used as an independent predictor of early AS and other cardiovascular events [45]. In terms of mechanism, OPG promoted AS by regulating lipid metabolism and the inflammatory response of vascular endothelial cells [46, 47], but there were no related reports on the role of OPG in VSMC differentiation. In the present study, we found that the expression of OPG gradually increased as the adventitial Sca-1^+^ progenitor cells differentiated into VSMCs, suggesting that OPG participates in VSMC differentiation. We found that knocking down OPG mimicked the inhibitory effect of miR-30c-5p on differentiation. These data were consistent with previous studies showing that miR-30c-5p played a protective role in AS, while OPG had a positive relationship with AS in clinical observational studies [25, 45]. Furthermore, we demonstrated that overexpression of miR-30c-5p inhibited OPG at the mRNA and protein levels, and that OPG was significantly upregulated after knocking down miR-30c-5p, suggesting that miR-30c-5p may regulate the expression of OPG solely by mRNA degradation or by both mRNA degradation and translation inhibition. The co-transfection assay showed that knocking down OPG aggravated the inhibitory effect of miR-30c-5p mimic on the VSMCs differentiation, while the promotional effect of the miR-30c-5p inhibitor on this process was weakened by knocking down OPG, further verifying the target regulation of OPG by miR-30c-5p. The dual-luciferase reporter gene assay provides the evidence to support that the mRNA 3′UTR of OPG is the direct target of miR-30c-5p. Some studies have shown that Sca-1^+^ progenitor cells also differentiated into osteoblasts and participated in vascular calcification. Taken together, the bone metabolism-related factor OPG was proposed for the first time to regulate the differentiation of adventitial Sca-1^+^ progenitor cells into VSMCs [48], which not only improves the bone vessel axis, but also suggests that there may be an intersection between the molecular mechanisms of AS and vascular calcification.

## Conclusions

This study demonstrates that miR-30c-5p expression is significantly decreased in atherosclerotic arteries and that miR-30c-5p inhibits the VSMC differentiation from adventitial Sca-1^+^ progenitor cells through targeting OPG, which may provide a new target for the treatment of VSMC-associated diseases.

## Data Availability

The datasets used and/or analyzed during the current study are available from the corresponding author on reasonable request.

## Abbreviations

miR-30c-5p: MicroRNA-30c-5p
OPG: Osteoprotegerin
AS: Atherosclerosis
VSMC: Vascular smooth muscle cell
Sca-1: Stem cell antigen-1
TGF-β1: Transforming growth factor-β1
siRNA: Small interfering RNA
SMαA: Smooth muscle α-actin
SM22α: Smooth muscle-22α
SMMHC: Smooth muscle myosin heavy chain
miR: MicroRNA
UTR: Untranslated region
EMT: Epithelial to mesenchymal transition
mut-3′UTR: Mutant 3′UTR
HA: Healthy artery
DA: Diseased artery
CM: Culture medium
FBS: Fetal bovine serum
MACS: Magnetic cell sorting system
DM: Differentiation medium
RT-qPCR: Real-time quantitative polymerase chain reaction
mGAPDH: Mouse GAPDH
HR: Heart rate
SBP: Systolic blood pressure
DBP: Diastolic blood pressure
CKD: Chronic kidney disease
BMI: Body mass index
TC: Total cholesterol
TG: Triglycerides
HDL: High-density lipoprotein
LDL: Low-density lipoprotein
VLDL: Very low-density lipoprotein
eGFR: Estimated glomerular filtration rate
SCr: Serum creatinine
ACEI: Angiotensin-converting enzyme inhibitor
ARB: Angiotensin-receptor blockers
PDGF-BB: Platelet derived growth factor BB
TNFRSF11B: Tumor necrosis factor receptor superfamily 11B
TRAIL: TNF-related apoptosis inducing ligand
